# Supplementary material for: The Evolution and Characterization of the RNA Interference Pathways in Lophotrochozoa
Source: Genome Biol Evol. 2024 May 7;16(5):evae098. doi: 10.1093/gbe/evae098 (PMC11114477; doi:10.1093/gbe/evae098)
Supplement: evae098_Supplementary_Data [file evae098_supplementary_data.zip › Supplementary_materials.zip!/selected_miRNAs/NoGe_selected_miRNAs/mirdeep_pdf_files/NMRB01002279.1_Notospermus_geniculatus_strain_Ushimado_scaffold2279,_whole_genome_shotgun_sequence_37110.pdf]

Provisional ID : NMRB01002279.1_Notospermus_geniculatus_strain_Ushimado_scaffold2279,_whole_genome_shotgun_sequence_37110
Score total : 1635.3
Score for star read(s) : 3.9
Score for read counts : 1627.5
Score for mfe : 0.9
Score for randfold : 0
Score for cons. seed : 3
Total read count : 3204
Mature read count : 3197
Loop read count : 0
Star read count : 7

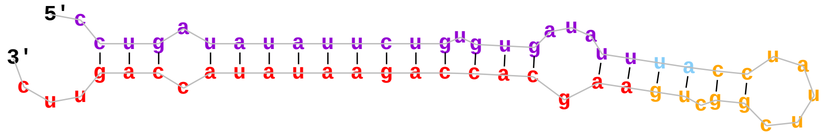

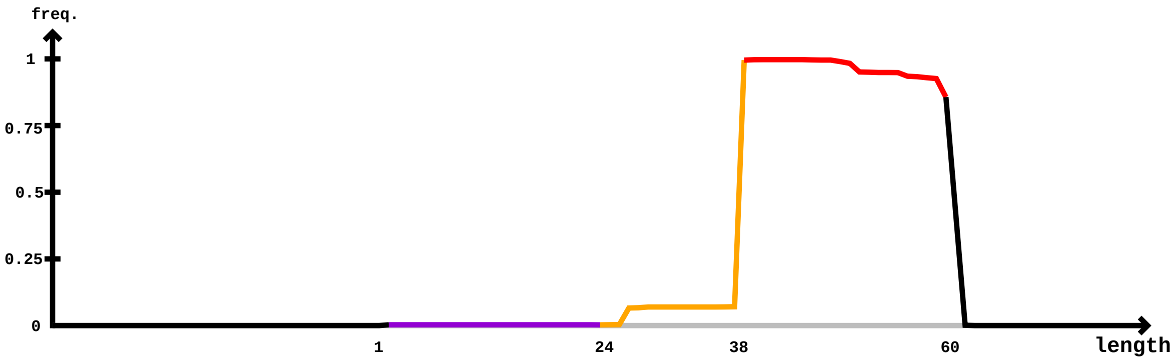

Star **Mature**

```
5'- gccugccgccguaguagcaugcaugucaugugaccugauauauucugugugauauuuaccuauucggcugaagcaccagaauauaccaguucauuugcuugcaaugaucauc  -3'   obs
    gccugccgccguaguagcaugcaugucaugugaccugauauauucugugugauauuuaccuauucggcugaagcaccagaauauaccaguucauuugcuugcaaugaucauc        exp
    ((((((....))))..)).((((.(.((.((((.(((.(((((((((.(((...((((((.....)).)))).))))))))))))).))).)))).))).))))........   reads   mm   sample
    ..........................................................ccuauucggcugaagcaccagaa...............................   5       0    Mm1
    ..........................................................ccuauucggcugaagcaccagaau..............................   5       0    Mm1
    ..........................................................ccuauucggcugaagcaccagaauUu............................   4       1    Mm1
    ..........................................................ccuauucggcugaagcaccagaauauacc.........................   3       0    Mm1
    ..........................................................ccuauucggcugaagcaAcagaauauacc.........................   1       1    Mm1
    ............................................................uauucggcugaagcaccagaauauaccaguuc....................   1       0    Mm1
    ......................................................................aagcaccagaauauaccagG......................   1       1    Mm1
    ......................................................................aagcaccagaauauaccagu......................   1       0    Mm1
    ......................................................................aagcaccagaauauaccGguu.....................   1       1    Mm1
    ......................................................................aagcaccagaauauaccaguu.....................   46      0    Mm1
    ......................................................................aagcaccagaUuauaccaguuc....................   2       1    Mm1
    ......................................................................aagcaccagaCuauaccaguuc....................   1       1    Mm1
    ......................................................................aagcaccagaauauaccaguuc....................   83      0    Mm1
    ......................................................................aagcaccagGauauaccaguuc....................   13      1    Mm1
    ......................................................................aagcaccagaauauaccGguuc....................   1       1    Mm1
    ......................................................................aagcaccagGauauaccaguuca...................   1       1    Mm1
    ......................................................................aagcaccagaauauaccaguuca...................   6       0    Mm1
    ......................................................................aagcaccagaauauaccaguucU...................   3       1    Mm1

    .................................ccugauauauucugugugauauu........................................................   4       0    Mm2
    .................................ccugauauauucugugugauauuu.......................................................   1       0    Mm2
    .................................ccugauauauucugugugauauuuaccuauucggcug..........................................   2       0    Mm2
    ........................................................uaccuauucggcugaagcacca..................................   2       0    Mm2
    ........................................................uaccuauucggcugaagcaccaga................................   4       0    Mm2
    ........................................................uaccuauucggcugaagcaccagU................................   1       1    Mm2
    ..........................................................ccuauucggcugaagcacc...................................   1       0    Mm2
    ..........................................................ccuauucggcugaagcaccaga................................   5       0    Mm2
    ..........................................................ccuauucggcugaagcUccagaa...............................   1       1    Mm2
    ..........................................................ccuauucggcugaagcaccagaa...............................   10      0    Mm2
    ..........................................................ccuauucggcugaagcaccagaau..............................   54      0    Mm2
    ..........................................................ccuauucggcugaagcaGcagaau..............................   1       1    Mm2
    ..........................................................ccuauucggcugaagcaccagaauU.............................   1       1    Mm2
    ..........................................................ccuauucggcugaagcaccagaaua.............................   2       0    Mm2
```

```
                                Star                             Mature
gccugccgccguaguagcaugcaugucaugugaccugauauauucugugugauauuuaccuauucggcugaagcaccagaauauaccaguucauuugcuugcaaugaucauc
..........................................................ccuauucggcugaagcaccagaauauacc.........................   15    0    Mm2
..........................................................ccuauucggcugaagcaccagaauauaccaguuc....................   7     0    Mm2
..........................................................ccuauucggcugaagcaccagaauauaccGguuc....................   1     1    Mm2
..........................................................ccuauucggcugaagcaAcagaauauaccaguuc....................   1     1    Mm2
...........................................................cuauucggcugaagcaccagaauauaccaguuc....................   2     0    Mm2
............................................................uauucggcugaagcaccagaauauaccag.......................   1     0    Mm2
............................................................uauucggcugaagcaccagaauauaccaguuc....................   8     0    Mm2
....................................................................ugaagcaccagaauauaccaguuc....................   1     0    Mm2
.....................................................................Caagcaccagaauauaccaguuc....................   1     1    Mm2
......................................................................aagcaccagaauauacca........................   1     0    Mm2
......................................................................aagcaccagaauauaccagu......................   1     0    Mm2
......................................................................aagcaccagaauauaccaguu.....................   80    0    Mm2
......................................................................aagcaccagaGuauaccaguu.....................   1     1    Mm2
......................................................................aagcaccagaUuauaccaguu.....................   1     1    Mm2
......................................................................aagcaccagGauauaccaguu.....................   1     1    Mm2
......................................................................aagcaccagaauauaccCguuc....................   2     1    Mm2
......................................................................aagcaccagUauauaccaguuc....................   2     1    Mm2
......................................................................aagcaccagaaCauaccaguuc....................   1     1    Mm2
......................................................................aagcaUcagaauauaccaguuc....................   1     1    Mm2
......................................................................aagcaNcagaauauaccaguuc....................   1     1    Mm2
......................................................................Nagcaccagaauauaccaguuc....................   1     1    Mm2
......................................................................aaUcaccagaauauaccaguuc....................   2     1    Mm2
......................................................................aagcaccUgaauauaccaguuc....................   1     1    Mm2
......................................................................aagcaccagaauauacUaguuc....................   8     1    Mm2
......................................................................aagcaccagaauauacGaguuc....................   1     1    Mm2
......................................................................aagcaccagaauauaccGguuc....................   3     1    Mm2
......................................................................aagcaccagaauauaccaguuG....................   1     1    Mm2
......................................................................aagcaccagaUuauaccaguuc....................   11    1    Mm2
......................................................................Cagcaccagaauauaccaguuc....................   1     1    Mm2
......................................................................aagcaccagaauaCaccaguuc....................   1     1    Mm2
......................................................................aagcaccagaauauacAaguuc....................   5     1    Mm2
......................................................................Uagcaccagaauauaccaguuc....................   1     1    Mm2
......................................................................aagcacUagaauauaccaguuc....................   1     1    Mm2
......................................................................aagcaccagaGuauaccaguuc....................   7     1    Mm2
......................................................................aagcaccagGauauaccaguuc....................   77    1    Mm2
......................................................................aagcaccagaauauaccUguuc....................   5     1    Mm2
......................................................................aagcaccagaauauaccaguuc....................   1695  0    Mm2
......................................................................aagcaccagCauauaccaguuc....................   1     1    Mm2
......................................................................aagcaccagaauauaccaguuU....................   4     1    Mm2
......................................................................aagcaccagGauauaccaguuca...................   5     1    Mm2
......................................................................aagcaccagaauauaccaguuca...................   37    0    Mm2
......................................................................aagcaccagaauauacUaguuca...................   1     1    Mm2
......................................................................aagcaccagaauauaccaguucU...................   37    1    Mm2
.......................................................................agcaccagaauauaccaguuc....................   4     0    Mm2

.................................ccugauauauucugugugauauuuaccuauucggcug..........................................   1     0    Fm1
......................................................................aagcaccagaauauaccaguu.....................   16    0    Fm1
......................................................................aagcaccagaauauaccagAu.....................   1     1    Fm1
......................................................................aagcaccagaauauaccaguuc....................   88    0    Fm1
......................................................................aagcaccagGauauaccaguuc....................   31    1    Fm1
......................................................................aagcaccagGauauaccaguuca...................   4     1    Fm1
......................................................................aagcaccagaauauaccaguuca...................   5     0    Fm1
......................................................................aagcaccagaUuauaccaguuca...................   1     1    Fm1

..........................................................ccuauucggcugaagcaAcagaau..............................   1     1    Fm2
..........................................................ccuauucggcugaagcaccagaau..............................   12    0    Fm2
..........................................................ccuauucggcugaagcaccagaauauacc.........................   14    0    Fm2
..........................................................ccuauucggcGgaagcaccagaauauacc.........................   1     1    Fm2
..........................................................ccuauucggcugaagcaccagaauaAacc.........................   1     1    Fm2
......................................................................aagcaccagaauauaccagu......................   3     0    Fm2
......................................................................aagcaccagaauauaccaguu.....................   22    0    Fm2
......................................................................aagcaccagaauauaccaguuA....................   18    1    Fm2
......................................................................aagcaccagaauauaccCguuc....................   1     1    Fm2
......................................................................aagcaccagaauauacAaguuc....................   2     1    Fm2
......................................................................aagcaccagaauauaccGguuc....................   1     1    Fm2
......................................................................aagcaccagGauauaccaguuc....................   19    1    Fm2
......................................................................aagcaccagaauauaccaguuc....................   175   0    Fm2
......................................................................aagcaccagaauauaccaguuca...................   50    0    Fm2
......................................................................aagcaccagGauauaccaguuca...................   1     1    Fm2
......................................................................aagcaccagaauauaccaguucU...................   17    1    Fm2
```

```
                                 Star                                 Mature
gccugccgccguaguagcaugcaugucaugugaccugauauauucugugugauauuuaccuauucggcugaagcaccagaauauaccaguucauuugcuugcaaugaucauc
......................................................................aagcaccagaauauaccaguucC...................   14    1   Fm2
......................................................................aagcaccagaauauacAaguuca...................   1     1   Fm2
......................................................................aagcaccagaUuauaccaguucau..................   1     1   Fm2

.................................ccugauauauucugugugauauu........................................................   1     0   Mm3
..........................................................ccuauucggcugaagcacc...................................   2     0   Mm3
..........................................................ccuauucggcugaagcaccaga................................   9     0   Mm3
..........................................................ccuauucggcugaagcaccagaa...............................   5     0   Mm3
..........................................................ccuauucggcugaagcaGcagaau..............................   1     1   Mm3
..........................................................ccuauucggcugaagcaccagaau..............................   24    0   Mm3
..........................................................ccuauucggcugaagcaccagaaA..............................   1     1   Mm3
..........................................................cGuauucggcugaagcaccagaau..............................   1     1   Mm3
..........................................................ccuauucggcugaagcaccagaauauac..........................   1     0   Mm3
..........................................................ccuauucggcugaagcaccagaauauacc.........................   12    0   Mm3
..........................................................ccuauucggcugaagcaccagaauauaccagu......................   1     0   Mm3
...........................................................cuauucggcugaagcaccagaauauaccaguuc....................   1     0   Mm3
.....................................................................Caagcaccagaauauaccaguuc....................   1     1   Mm3
......................................................................aagcaccagaauauaccaguu.....................   23    0   Mm3
......................................................................aagcaccagaauauaccGguu.....................   1     1   Mm3
......................................................................aagcaccagaauauaccaguA.....................   1     1   Mm3
......................................................................aagcaccagaauauaccaguuU....................   1     1   Mm3
......................................................................aagcaccagaauauacAaguuc....................   1     1   Mm3
......................................................................aagcaccagaauauacGaguuc....................   1     1   Mm3
......................................................................aagcaccagaGuauaccaguuc....................   1     1   Mm3
......................................................................aagcaccagaauauaccUguuc....................   1     1   Mm3
......................................................................aagcaccagaauauaccGguuc....................   2     1   Mm3
......................................................................aagcaccagaauauaccCguuc....................   1     1   Mm3
......................................................................aagcaccagaauauaccaguuA....................   1     1   Mm3
......................................................................aagcaccagaauauaccaguuc....................   355   0   Mm3
......................................................................aagcaccagaauaAaccaguuc....................   1     1   Mm3
......................................................................aagUaccagaauauaccaguuc....................   1     1   Mm3
......................................................................aagcaccagUauauaccaguuc....................   1     1   Mm3
......................................................................aagcaccagGauauaccaguuc....................   14    1   Mm3
......................................................................aagcaccagaCuauaccaguuc....................   1     1   Mm3
......................................................................aagcaccagaauauaccaguucC...................   1     1   Mm3
......................................................................aagcaccagaauauaccaguuca...................   7     0   Mm3
......................................................................aagcaccagaauauaccaguucU...................   18    1   Mm3
......................................................................aagcaccagGauauaccaguuca...................   2     1   Mm3
......................................................................aagcaccagaauauaccaguucau..................   2     0   Mm3
......................................................................aagcaccagaauauaccaguucUu..................   1     1   Mm3
.......................................................................agcaccagaauauaccaguuc....................   1     0   Mm3
........................................................................gcaccagaauauaccaguuc....................   1     0   Mm3

.................................ccugauauauucugugCgauau.........................................................   1     1   Fm3
........................................................uaccuauucggcugaagcaccagaa...............................   2     0   Fm3
..........................................................ccuauucggcugaagcaccaga................................   1     0   Fm3
..........................................................ccuauucggcugaagcaccagaau..............................   11    0   Fm3
......................................................................aagcaccagaauauacca........................   5     0   Fm3
......................................................................aagcaccagaauauaccag.......................   12    0   Fm3
......................................................................aagcaccagaauauaccagu......................   3     0   Fm3
......................................................................aagcaccagaauauaccagG......................   1     1   Fm3
......................................................................aagcaccagaGuauaccaguu.....................   1     1   Fm3
......................................................................aagcaccagaaCauaccaguu.....................   1     1   Fm3
......................................................................aagcaccagaauauaccUguu.....................   1     1   Fm3
......................................................................aagcaccagaauauaccaguu.....................   41    0   Fm3
......................................................................aagcaccagGauauaccaguu.....................   2     1   Fm3
......................................................................aagcaccagaauauaccaguuc....................   39    0   Fm3
......................................................................aagcaccagaauauaccaguuA....................   3     1   Fm3
......................................................................aagcaccagGauauaccaguuc....................   9     1   Fm3
......................................................................aagcaccagaauauaccaguuca...................   2     0   Fm3
......................................................................aagcaccagGauauaccaguuca...................   2     1   Fm3
```
